# Significant association of serum carotenoids with the systemic immune-inflammation index: A cross-sectional study based on NHANES

**DOI:** 10.1097/MD.0000000000042942

**Published:** 2025-06-20

**Authors:** Hongcun Sun, Jiandao Hu, Wenbo Jiang

**Affiliations:** a Department of Otorhinolaryngology-Head and Neck Surgery, The Affiliated People’s Hospital of Ningbo University, Ningbo, China.

**Keywords:** carotenoids, inflammation, NHANES, systemic immune-inflammation index

## Abstract

The systemic immune-inflammation index (SII) is an emerging inflammatory marker. Carotenoids have anti-inflammatory properties. Therefore, this study aims to explore the association of serum carotenoids with SII. This cross-sectional study analyzed data from the 2001 to 2006 and 2017 to 2018 cycles of National Health and Nutrition Examination Survey. Multivariate linear regression models were employed to evaluate the relationship. Restricted cubic splines models were used to assess potential nonlinear relationships. Subgroup analyses and sensitivity analysis were also performed. Our study included 11,914 eligible participants. After adjusting for all covariates, the total carotenoids levels showed a negative correlation with SII (*P* < .001). Furthermore, the levels of α-carotene, β-carotene, β-cryptoxanthin, and lutein/zeaxanthin still had significant negative associations (all *P* < .001). Specifically, individuals in the highest quartile of α-carotene, β-carotene, β-cryptoxanthin, lutein/zeaxanthin, and lycopene had lower SII levels compared to those in the lowest quartile (all *P* < .05). Nonlinear relationships were observed between total carotenoids, α-carotene, β-carotene, β-cryptoxanthin, lutein/zeaxanthin, and SII (all *P* < .05), while a linear relationship between lycopene and SII was found (*P* = .070). Subgroup analyses and sensitivity analysis indicated that the results were robust. This study reveals a significant negative association between serum carotenoids and SII, highlighting the potential anti-inflammatory role of carotenoids.

## 1. Introduction

Inflammation is a defensive response to various harmful stimuli. While acute inflammation is a protective mechanism, chronic inflammation can be more detrimental, increasing the risk of cellular damage and contributing to the development of chronic diseases. Numerous studies have demonstrated that chronic inflammation is closely linked to the onset and progression of systemic diseases, including cardiovascular diseases, metabolic disorders, cancers, and neurodegenerative diseases.^[1–4]^ The systemic immune-inflammation index (SII), an emerging inflammatory marker, has gained significant attention in recent years. SII can provide a comprehensive reflection of the body’s inflammatory state by integrating the roles of platelets, neutrophils, and lymphocytes in immune-inflammatory responses. Epidemiological evidence suggests that elevated SII levels are associated with an increased risk of conditions such as psoriasis, hearing loss, and cancers.^[5–8]^ Additionally, SII has been shown to predict the prognosis of sudden hearing loss, nasal polyps, and various cancers.^[9–15]^ Moreover, SII plays a critical role in monitoring drug efficacy and guiding treatment strategies.^[16]^ In summary, SII serves as a valuable marker for the diagnosis, prognosis, and treatment evaluation of numerous diseases.

Carotenoids, a group of antioxidant pigments found in fruits, vegetables, and seaweed, primarily include α-carotene, β-carotene, β-cryptoxanthin, lutein/zeaxanthin, and lycopene.^[17]^ Serum carotenoid levels are inversely associated with the risk of numerous chronic diseases, such as metabolic syndrome, cancers, cardiovascular diseases, cerebrovascular disorders, and aging-related conditions.^[18–26]^ Moreover, growing evidence over the past few decades has highlighted the protective role of carotenoids in neurodegenerative diseases.^[27]^ In epidemiological studies, carotenoids are recognized not only for their antioxidant properties but also for their anti-inflammatory effects.^[28,29]^ They can stimulate the proliferation of B- and T-lymphocytes and enhance macrophage activity, thereby boosting immune function. These immune cells play a vital role in identifying and eliminating pathogens.^[30]^ Additionally, carotenoids inhibit the production of inflammatory cytokines, such as interleukin-6 and tumor necrosis factor α, which are key mediators of inflammatory responses.^[31,32]^ By reducing these inflammatory markers, carotenoids help mitigate inflammation and maintain immune balance. Beyond their direct effects on immune cells, carotenoids also indirectly support immune function by enhancing the body’s antioxidant capacity. Oxidative stress, caused by excessive free radicals, can damage cellular structures, trigger inflammation, and impair immune system function. Carotenoids counteract this by neutralizing free radicals, thereby protecting cells and promoting overall immune health.

Theoretically, carotenoids may improve inflammatory states. However, the specific relationship between carotenoids and the SII remains underexplored. A deeper understanding of this association is crucial for elucidating the pathogenesis of chronic diseases and identifying potential preventive and therapeutic targets. Leveraging the widely representative National Health and Nutrition Examination Survey (NHANES) database in the United States, this study aims to comprehensively analyze the relationship between serum carotenoids and SII in the adult population. The findings could provide new insights into the early prevention of chronic diseases, personalized diagnosis and treatment, and the development of precise nutritional intervention strategies.

## 2. Materials and methods

### 2.1. Study design and population

**Figure 1.**
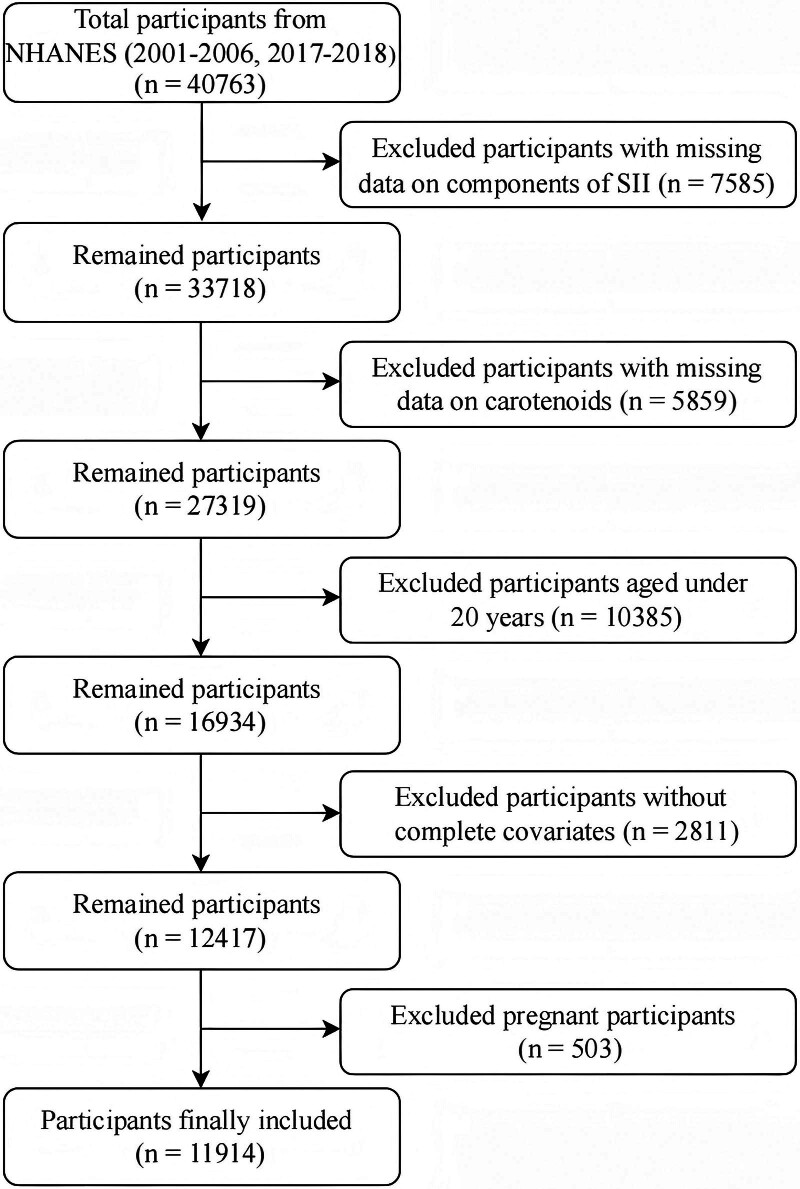
Flow diagram of participant selection. NHANES = National Health and Nutrition Examination Survey, SII = systemic immune-inflammation index.

The NHANES database is an authoritative, multi-stage, stratified, and highly representative large-scale research project designed to accurately assess the health and nutritional status of adults and children in the United States. For this study, data were extracted from 4 cycles of NHANES (2001–2006 and 2017–2018) to analyze serum carotenoid levels. This selection ensures that the findings reflect both long-term trends and recent changes. Initially, 40,763 adults were considered. To ensure sample homogeneity and data reliability, strict exclusion criteria were applied. Participants with incomplete data on serum carotenoids or components of SII, those under 20 years of age, individuals with missing covariate values, and pregnant women were excluded. Ultimately, 11,914 participants were included in the study (Fig. 1). The Ethics Review Board of the National Center for Health Statistics (NCHS) approved all NHANES protocols, and all participants signed a written informed consent form before participation. In addition, NCHS gave approval for public dissemination. All additional materials can be downloaded at https://wwwn.cdc.gov/nchs/nhanes.

### 2.2. Assessment of serum carotenoids

The NHANES documentation provides detailed descriptions of the laboratory techniques used to measure serum carotenoids. These techniques employ multi-wavelength photodiode-array absorbance detection combined with high-performance liquid chromatography. The quantification of analytes is based on external standard quantitation, where response factors are determined using standard solutions, with the peak area of the analyte at 450 nm serving as the basis for calculation.

Research indicates that over 95% of carotenoids in human serum consist of 6 major types: α-carotene, β-carotene, β-cryptoxanthin, lycopene, and the combined lutein/zeaxanthin.^[33]^ Therefore, total serum carotenoids were calculated as the sum of these 6 components.^[34]^ In this study, serum carotenoids were treated as exposure variables.

### 2.3. Assessment of SII

Peripheral blood samples from NHANES participants were analyzed at Mobile Examination Centers using a Beckman Coulter HMX Hematology Analyzer. Lymphocyte, neutrophil, and platelet counts were measured through complete blood count and expressed as × 10⁹/L. The SII was calculated using the formula: platelet count × neutrophil count/ lymphocyte count (in units of 10⁹/L).^[35]^ In this study, SII was treated as the outcome variable.

### 2.4. Covariates

Thirteen covariates were included in our study. The covariates collected were as follows: gender (male or female), age, race (Mexican American, other Hispanic, non-Hispanic white, non-Hispanic black, or other race), education level (less than high school, high school, or more than high school), marital status (married/living with a partner or living alone), and general health condition (good, fair, or poor). The poverty income ratio (PIR), defined as the ratio of total household income to the poverty threshold, was categorized into 3 groups: ≤ 1.30, 1.31 to 3.50, and > 3.50. Body mass index (BMI), calculated as weight (kg) divided by height squared (m²), was classified into 3 categories: < 25, 25 to 29.9, and ≥ 30. Additional binary variables included smoking status (having smoked at least 100 cigarettes in life), alcohol use (consuming 5 or more drinks daily), vigorous activity (large increases in breathing or heart rate for at least 10 minutes continuously), and moderate activity (small increases in breathing or heart rate for at least 10 minutes continuously). Data on C-reactive protein (CRP) levels (mg/L) were also collected. Detailed methods for acquiring and analyzing these variables are available at https://wwwn.cdc.gov/nchs/nhanes/.

### 2.5. Statistical analysis

In our study, the median value of total carotenoids was considered as the cutoff for the 2 groups (low and high group) to describe baseline characteristics of participants. The quartiles of serum carotenoid levels (α-carotene, β-carotene, β-cryptoxanthin, lycopene, and the combined lutein/zeaxanthin) were determined based on the distribution within the study population. Categorical variables were presented as numbers (percentages), and continuous variables with a normal distribution were expressed as mean ± standard deviation. The chi-square test was used to compare categorical variables between groups, while the *t* test was applied for continuous variables. Linear regression analysis was performed to assess trends and investigate the relationship between serum carotenoids, serum carotenoids quartiles, and SII. Multivariable linear regression analysis was conducted using 3 models to evaluate the impact of covariates on this relationship. Model 1 included no covariates. Model 2 was adjusted for gender, age, race, education level, PIR, and marital status. Model 3 was further adjusted for additional covariates, including smoking, alcohol use, BMI, CRP, general health condition, vigorous activity, and moderate activity.

To visualize the relationship between serum carotenoids and SII and explore potential nonlinear associations, restricted cubic spline models (RCS) were employed. Subgroup analysis was performed to minimize potential biases. Sensitivity analysis using multiple imputation for missing covariate values was conducted to confirm the robustness of the findings after excluding missing data for SII and the 6 carotenoids.

Data processing and analysis were performed using R version 4.4.0 and Zstats 1.0 (www.zstats.net), except for multiple imputation, which was conducted using SPSS (version 26.0). A *P*-value < .05 was considered statistically significant.

## 3. Results

### 3.1. Baseline characteristics of the study population

Table 1 presents the baseline characteristics and distribution of participants between the 2 groups. A total of 11,914 participants were included in this study, with a median serum total carotenoid level of 64.29 µg/dL. Compared to the low total carotenoid group, the high total carotenoid group was older and had a significantly higher proportion of males, non-Hispanic Whites, individuals who were married or living with a partner, those with more than a high school education, a PIR > 3.50, and a BMI of 25 to 29.9 (all *P* < .001). Additionally, the high total carotenoid group reported better general health (*P* < .001). In contrast, the high total carotenoid group had a lower prevalence of smoking, alcohol use, and vigorous activity but a higher prevalence of moderate activity (all *P* < .001). Comparisons of inflammatory-related variables revealed that the high total carotenoid group had significantly lower levels of SII, neutrophils, platelets, and CRP (all *P* < .001). Similarly, lymphocyte levels were lower in the high total carotenoid group (*P* = .013).

**Table 1:** Basic characteristics of the study population.

Variables	Total (n = 11914)	Total carotenoids level		*p*
		Low (n = 5957)	High (n = 5957)	
Age (years)	50.30 ± 17.94	49.74 ± 18.15	50.85 ± 17.72	< .001
SII (10^9^/L)	575.48 ± 370.58	608.37 ± 415.20	542.59 ± 316.43	< .001
Lymphocyte (10^9^/L)	2.17 ± 1.62	2.21 ± 1.42	2.14 ± 1.80	.013
Neutrophil (10^9^/L)	4.24 ± 1.63	4.48 ± 1.72	4.00 ± 1.50	< .001
Peripheral platelet 10^9^/L)	262.39 ± 70.04	266.11 ± 74.59	258.68 ± 64.97	< .001
CRP (mg/L)	1.26 ± 3.80	1.59 ± 4.64	0.92 ± 2.68	< .001
Gender				< .001
Male	6485 (54.43)	3356 (56.34)	3129 (52.53)	
Female	5429 (45.57)	2601 (43.66)	2828 (47.47)	
PIR				< .001
≤ 1.300	2974 (24.96)	1752 (29.41)	1222 (20.51)	
1.301–3.500	4738 (39.77)	2472 (41.50)	2266 (38.04)	
> 3.500	4202 (35.27)	1733 (29.09)	2469 (41.45)	
Race				< .001
Mexican American	2191 (18.39)	942 (15.81)	1249 (20.97)	
Other Hispanic	529 (4.44)	233 (3.91)	296 (4.97)	
Non-Hispanic White	6159 (51.70)	3296 (55.33)	2863 (48.06)	
Non-Hispanic Black	2302 (19.32)	1212 (20.35)	1090 (18.30)	
Other Race	733 (6.15)	274 (4.60)	459 (7.71)	
Marital status				< .001
Married or living with partner	7484 (62.82)	3555 (59.68)	3929 (65.96)	
Living alone	4430 (37.18)	2402 (40.32)	2028 (34.04)	
Educational level				< .001
Less than high school	2896 (24.31)	1564 (26.25)	1332 (22.36)	
High school	2899 (24.33)	1643 (27.58)	1256 (21.08)	
More than high school	6119 (51.36)	2750 (46.16)	3369 (56.56)	
BMI(kg/m²)				< .001
≥ 30	4169 (34.99)	2520 (42.30)	1649 (27.68)	
25–29.9	4133 (34.69)	1880 (31.56)	2253 (37.82)	
< 25	3612 (30.32)	1557 (26.14)	2055 (34.50)	
General health condition				< .001
Good	9286 (77.94)	4395 (73.78)	4891 (82.11)	
Fair	2247 (18.86)	1286 (21.59)	961 (16.13)	
Poor	381 (3.20)	276 (4.63)	105 (1.76)	
Smoking (yes)	6375 (53.51)	3665 (61.52)	2710 (45.49)	< .001
Alcohol use (yes)	1969 (16.53)	1186 (19.91)	783 (13.14)	< .001
Vigorous activity (yes)	3542 (29.73)	1430 (24.01)	2112 (35.45)	< .001
Moderate activity (yes)	5973 (50.13)	2687 (45.11)	3286 (55.16)	< .001

Mean ± standard deviation for continuous variables: the *P*-value was calculated by a t test. Numbers (percentages) for categorical variables: the *P*-value was calculated by a chi-square test.

BMI = body mass index, CRP = C-reactive protein, PIR = poverty income ratio, SII = systemic immune-inflammation index.

### 3.2. Association of serum carotenoids with SII

Table 2 shows the linear regression results examining the relationship between serum carotenoids and SII. Univariate analysis revealed that higher total carotenoid levels were associated with lower SII levels (*P* < .001). Specifically, α-carotene, β-carotene, β-cryptoxanthin, lutein/zeaxanthin, and lycopene showed significant associations (all *P* < .001). After adjusting for confounders in Models 2 and 3, total carotenoids, α-carotene, β-carotene, β-cryptoxanthin, and lutein/zeaxanthin maintained significant negative associations with SII levels (all *P* < .05). However, no significant association was observed between serum lycopene and SII levels in Model 3 (*P* = .138).

**Table 2:** Association of serum carotenoids with SII was analyzed by multivariable linear regression.

Variables	Model 1		Model 2		Model 3	
	β (95% CI)	*P*	β (95% CI)	*P*	β (95% CI)	*P*
Total carotenoids	−0.80 (−0.96 ~ −0.65)	< .001	−0.79 (−0.94 ~ −0.63)	< .001	−0.66 (−0.83 ~ −0.50)	< .001
α-Carotene	−3.84 (−4.85 ~ −2.84)	< .001	−4.01 (−5.01 ~ −3.00)	< .001	−3.44 (−4.46 ~ −2.42)	< .001
β-Carotene	−0.95 (−1.23 ~ −0.66)	< .001	−1.20 (−1.49 ~ −0.90)	< .001	−1.01 (−1.32 ~ −0.71)	< .001
β-Cryptoxanthin	−2.76 (−3.51 ~ −2.02)	< .001	−2.36 (−3.14 ~ −1.58)	< .001	−1.72 (−2.52 ~ −0.93)	< .001
Lutein/zeaxanthin	−3.27 (−3.87 ~ −2.68)	< .001	−2.75 (−3.36 ~ −2.15)	< .001	−2.46 (−3.08 ~ −1.85)	< .001
Lycopene	−1.35 (−1.96 ~ −0.73)	< .001	−0.93 (−1.57 ~ −0.30)	.004	−0.48 (−1.11 ~ 0.15)	.138

CI = confidence interval, SII = systemic immune-inflammation index.

Model 1: No variables was adjusted.

Model 2: Gender, age, race, educational level, PIR, and marital status were adjusted.

Model 3: All variables in Model 2 plus general health condition, BMI, smoking, alcohol use, vigorous activity, moderate activity, and CRP were adjusted.

### 3.3. Relationship of different quartiles of serum carotenoids with SII

Table 3 presents the multivariable regression analysis of serum carotenoid quartiles and SII. Participants in the fourth (Q4) and third (Q3) quartiles of α-carotene, β-carotene, β-cryptoxanthin, and lutein/zeaxanthin exhibited significantly lower SII levels compared to those in the first quartile (Q1) across all 3 models (*P* < .05). Similarly, participants in the second quartile (Q2) of β-cryptoxanthin and lutein/zeaxanthin also showed lower SII levels than those in Q1 across all models (*P* < .05). For lycopene, participants in Q4, Q3, and Q2 had lower SII levels than those in Q1 in Models 1 and 2 (*P* < .05), while only Q4 and Q2 showed lower SII levels in Model 3 (*P* < .05). As the concentrations of α-carotene, β-carotene, β-cryptoxanthin, and lutein/zeaxanthin increased, SII levels decreased, with all models showing a significant trend (*P *< .05). However, a significant trend between serum lycopene quartiles and SII levels was only observed in Models 1 and 2 (*P* < .05).

**Table 3:** Association of serum carotenoids quartile with SII was analyzed by multivariable linear regression.

Variables	Model 1		Model 2		Model 3	
	β (95% CI)	*P*	β (95% CI)	*P*	β (95% CI)	*P*
α-Carotene quartile						
Q1 (0.21–1.39) ug/dL	reference		reference		reference	
Q2 (1.40–2.69) ug/dL	−4.11 (−23.03 ~ 14.81)	.670	−16.65 (−35.53 ~ 2.22)	.084	−7.86 (−26.69 ~ 10.98)	.414
Q3 (2.70–5.19) ug/dL	−29.80 (−48.58 ~ −11.03)	.002	−47.78 (−66.97 ~ −28.59)	< .001	−33.55 (−52.92 ~ −14.18)	< .001
Q4 (5.20–205.00) ug/dL	−59.37 (−78.20 ~ −40.54)	< .001	−82.29 (−102.06 ~ −62.52)	< .001	−64.87 (−85.30 ~ −44.44)	< .001
*P* for trend	< 0.001		< 0.001		< 0.001	
β-Carotene quartile						
Q1 (0.64–7.39) ug/dL	reference		reference		reference	
Q2 (7.40–12.99) ug/dL	−13.92 (−32.71 ~ 4.86)	.146	−19.75 (−38.29 ~ −1.21)	.037	−11.99 (−30.51 ~ 6.53)	.204
Q3 (13.00–23.49) ug/dL	−36.82 (−55.60 ~ −18.03)	< .001	−48.01 (−66.85 ~ −29.18)	< .001	−34.68 (−53.77 ~ −15.59)	< .001
Q4 (23.50–411.47) ug/dL	−67.84 (−86.60 ~ −49.08)	< .001	−89.64 (−109.16 ~ −70.12)	< .001	−73.66 (−93.93 ~ −53.40)	< .001
*P* for trend	< 0.001		< 0.001		< 0.001	
β-Cryptoxanthin quartile						
Q1 (0.14–4.59) ug/dL	reference		reference		reference	
Q2 (4.60–7.42) ug/dL	−46.11 (−64.87 ~ −27.35)	< .001	−34.19 (−52.78 ~ −15.61)	< .001	−20.62 (−39.23 ~ −2.01)	.030
Q3 (7.43–12.29) ug/dL	−54.12 (−72.92 ~ −35.32)	< .001	−39.00 (−57.87 ~ −20.14)	< .001	−20.70 (−39.83 ~ −1.57)	.034
Q4 (12.30–150.80) ug/dL	−85.37 (−104.14 ~ −66.60)	< .001	−69.36 (−89.00 ~ −49.71)	< .001	−47.92 (−68.20 ~ −27.64)	< .001
*P* for trend	< 0.001		< 0.001		< 0.001	
Lutein/zeaxanthin quartile						
Q1 (0.14–10.69) ug/dL	reference		reference		reference	
Q2 (10.70–14.99) ug/dL	−51.13 (−69.88 ~ −32.38)	< .001	−37.40 (−56.03 ~ −18.78)	< .001	−28.10 (−46.70 ~ −9.50)	.003
Q3 (15.00–21.09) ug/dL	−80.66 (−99.33 ~ −62.00)	< .001	−58.82 (−77.63 ~ −40.00)	< .001	−47.81 (−66.71 ~ −28.91)	< .001
Q4 (21.10–351.00) ug/dL	−120.80 (−139.48 ~ −102.12)	< .001	−97.37 (−116.62 ~ −78.11)	< .001	−82.71 (−102.32 ~ −63.09)	< .001
*P* for trend	< 0.001		< 0.001		< 0.001	
Lycopene quartile						
Q1 (0.21–13.89) ug/dL	reference		reference		reference	
Q2 (13.90–20.29) ug/dL	−32.93 (−51.76 ~ −14.11)	< .001	−31.85 (−50.67 ~ −13.04)	< .001	−24.48 (−43.19 ~ −5.78)	.010
Q3 (20.30–28.09) ug/dL	−29.04 (−47.86 ~ −10.21)	.003	−25.71 (−44.83 ~ −6.59)	.008	−15.84 (−34.88 ~ 3.19)	.103
Q4 (28.10–80.600) ug/dL	−45.41 (−64.24 ~ −26.57)	< .001	−36.86 (−56.31 ~ −17.42)	< .001	−23.19 (−42.59 ~ −3.78)	.019
*P* for trend	< 0.001		0.001		0.065	

CI = confidence interval, SII = systemic immune-inflammation index.

Model 1: No variables was adjusted.

Model 2: Gender, age, race, educational level, PIR, and marital status were adjusted.

Model 3: All variables in Model 2 plus general health condition, BMI, smoking, alcohol use, vigorous activity, moderate activity, and CRP were adjusted.

### 3.4. Subgroup analyses

**Figure 2.**
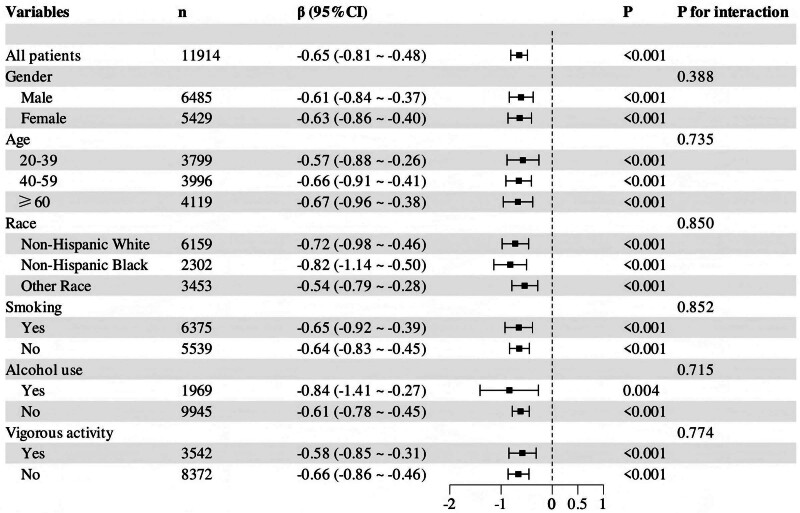
Forest plot showing associations between total carotenoids and SII by gender, age, race, and smoking, alcohol use, and vigorous activity in Model 3. SII = systemic immune-inflammation index.

Further subgroup analyses were performed based on age (20–39 years, 40–59 years, and ≥ 60 years), gender (male and female), race (non-Hispanic White, non-Hispanic Black, and other races), smoking status (yes or no), alcohol use (yes or no), and vigorous activity (yes or no). As shown in Figure 2, the association between total serum carotenoids and SII was consistent across all subgroups, with no significant interactions observed (all *P* > .05).

### 3.5. The nonlinear relationship between serum carotenoids and SII

**Figure 3.**
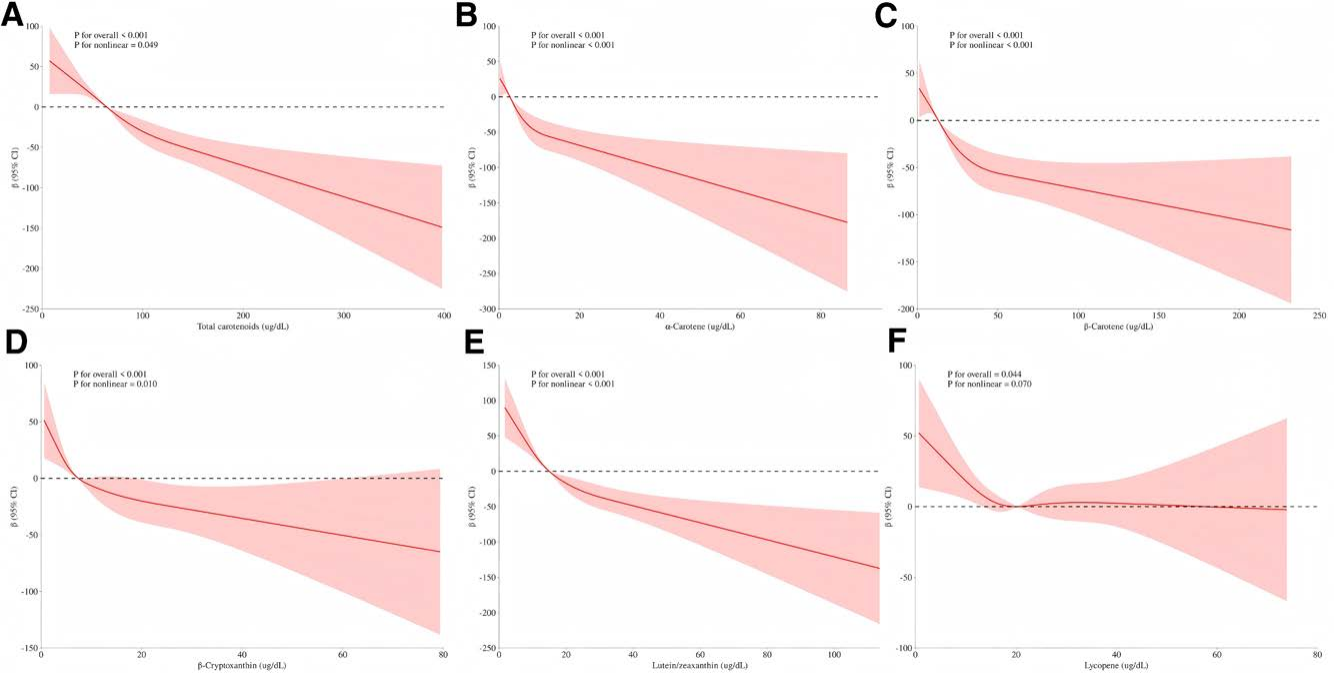
RCS models showing the relationship between serum carotenoids and SII in Model 3. RCS = restricted cubic spline, SII = systemic immune-inflammation index.

Figure 3 illustrates the nonlinear relationship between serum carotenoids and SII using RCS models. After adjusting for confounding factors in Model 3, a nonlinear relationship was observed between total carotenoids and SII (*P* < .001). Specifically, nonlinear associations were identified for α-carotene, β-carotene, β-cryptoxanthin, and lutein/zeaxanthin with SII (all *P* < .05). In contrast, lycopene showed a linear relationship with SII (*P *= .070). As shown in Table 4, there was a threshold effect in the association between total carotenoids and SII (*P* = .031). When total carotenoids levels were lower than 147.574 ug/dL, there was a negative association between total carotenoids and SII (*P* < .001). However, no association was found when total carotenoids levels were higher than 147.574 ug/dL (*P* = .979).

**Table 4:** The threshold effect analysis in the association between total carotenoids and SII.

Outcome	Effect	*P*
Model 1 Fitting model by standard linear regression	−0.66 (−0.82 ~ −0.50)	<.001
Model 2 Fitting model by 2-piecewise linear regression		
Inflection point	147.574	
< 147.574	−0.82 (−1.08 ~ −0.57)	<.001
≥ 147.574	−0.00 (−0.36 ~ 0.35)	.979
P for likelihood test		.031

### 3.6. Sensitivity analysis

In our study, sensitivity analysis ensured conclusion robustness. After eliminating missing data of carotenoids and SII and those pregnant participants, we performed multiple imputation for missing values of all covariates (Table S1, Supplemental Digital Content, https://links.lww.com/MD/P227). After adjusting for confounding factors in Model 3, total carotenoids, α-carotene, β-carotene, β-cryptoxanthin, and lutein/zeaxanthin still had significant negative associations with SII (all *P* < .001). However, no association was found between serum lycopene and SII (*P* = .075).

## 4. Discussion

Through an in-depth analysis of data from the NHANES, this study is the first to reveal a significant negative correlation between serum carotenoids and SII. Subgroup analyses and interaction tests further demonstrated that this association was consistent across different populations. Additionally, a nonlinear relationship between serum carotenoids and SII was identified. Specifically, nonlinear associations were observed for total carotenoids, α-carotene, β-carotene, β-cryptoxanthin, and lutein/zeaxanthin with SII. In contrast, lycopene exhibited a linear relationship with SII. Sensitivity analysis confirmed the robustness of these findings.

Several previous studies provide strong evidence supporting our results. For example, 1 study demonstrated an inverse relationship between serum carotenoids (including total levels of 4 carotenoids and individual carotenoids such as α-carotene, β-carotene, zeaxanthin/lutein, and β-cryptoxanthin) and leukocyte counts. In contrast, circulating lycopene showed no significant association with leukocyte count.^[36]^ Another community-based cross-sectional study conducted in China examined the association between serum carotenoids and high-sensitivity CRP.^[32]^ The results revealed negative associations between 5 major serum carotenoids (α-carotene, β-carotene, β-cryptoxanthin, lycopene, and lutein/zeaxanthin, as well as total carotenoid levels) and high-sensitivity CRP. Recent studies have further highlighted a more pronounced negative correlation between lutein/zeaxanthin and CRP levels.^[37]^ These findings underscore the close relationship between carotenoids and inflammation, offering new perspectives for improving inflammatory conditions.

The mechanisms by which carotenoids regulate SII levels are complex and primarily involve 2 key aspects. The first mechanism is the inhibition of inflammatory cell infiltration.^[38]^ Carotenoids can suppress the infiltration of inflammatory cells, such as macrophages and neutrophils, thereby reducing tissue damage. They also inhibit the production of chemokines, preventing the migration of inflammatory cells to sites of inflammation. Additionally, carotenoids modulate intercellular adhesion molecules, weakening the interaction between inflammatory cells and vascular endothelial cells. The second mechanism involves the modulation of inflammatory cytokine expression. Carotenoids can inhibit the release of pro-inflammatory cytokines while promoting the production of anti-inflammatory cytokines, thereby balancing the inflammatory response.^[31,39]^ This cytokine balance helps prevent immune system overactivation, avoids uncontrolled inflammatory responses, and maintains immune homeostasis. Ultimately, these effects are reflected in a mitigated inflammatory state, as indicated by SII. Carotenoids also target key inflammatory signaling pathways, such as nuclear factor kappa B and nuclear factor erythroid 2-related factor 2, blocking the transcription of pro-inflammatory cytokines.^[40,41]^

The findings of this study have significant implications for clinical practice, offering valuable insights for early disease diagnosis, risk assessment, and the selection of therapeutic targets. In terms of early disease diagnosis, serum carotenoids could be incorporated into routine testing as a potential biomarker. Given their close association with SII, low levels of serum carotenoids combined with an upward trend in SII may indicate a pre-inflammatory stress state. This could alert clinicians to further investigate the risk of chronic diseases, such as cardiovascular diseases, diabetes, and cancers. For individuals with high-risk factors, such as a family history of disease or unhealthy lifestyles, regular monitoring of serum carotenoid levels alongside SII could help detect subtle physiological changes before the onset of clinical symptoms. This enables early intervention and provides valuable time for precise treatment. From the perspective of disease risk assessment, the relationship between serum carotenoids and SII offers a strong foundation for constructing comprehensive risk prediction models. Clinicians could integrate serum carotenoids as an independent risk factor into existing risk assessment systems, alongside traditional cardiovascular risk factors (e.g., blood lipids, blood pressure, and blood glucose), inflammatory markers (e.g., CRP), and lifestyle factors. This integration would allow for more accurate predictions of an individual’s likelihood of developing chronic diseases over a specific period. In the exploration of therapeutic targets, this study highlights the potential for developing new drugs and optimizing existing treatment strategies. Given that carotenoids can regulate the body’s inflammatory response through multiple pathways, such as antioxidant and immune-modulating actions, novel anti-inflammatory drugs based on carotenoid structures or mechanisms could become powerful tools for treating inflammation-related diseases. Furthermore, for patients undergoing anti-inflammatory therapy, monitoring changes in serum carotenoid levels could serve as an indirect indicator of treatment efficacy, guiding clinicians in adjusting drug dosages or treatment plans to achieve optimal outcomes.

This study has several notable strengths. First, it leverages the large and highly representative NHANES database, which includes a diverse sample of adults from various regions, races, socioeconomic backgrounds, and lifestyles across the United States. This ensures that the study results accurately reflect the true conditions of the U.S. adult population and possess high external validity, providing a valuable reference for related studies worldwide. Second, the study incorporates a wide range of covariates, including demographic information, lifestyle factors, and physical activity levels. By thoroughly examining the potential confounding effects of these variables, the study maximizes the clarity of the core association between serum carotenoids and SII, enhancing the reliability and precision of its conclusions. Third, the study employs advanced and rigorous statistical methods, such as multivariate linear regression, subgroup analyses, RCS analysis, and sensitivity analysis. These methods explore the relationships from multiple dimensions, capture potential nonlinear trends, and rigorously validate the robustness of the results, providing solid data support for the study’s conclusions.

However, this study also has certain limitations. First, its cross-sectional design limits the ability to establish causal relationships between serum carotenoids and SII. Further validation through prospective cohort studies or interventional trials is needed to clarify the underlying causal mechanisms. Second, despite adjusting for numerous covariates, the complexity of real-world situations means that unmeasured or difficult-to-quantify factors, such as environmental pollutant exposure and gut microbiota differences, may still influence the results. These factors could subtly affect the relationship between serum carotenoids and SII, potentially introducing some degree of bias. Third, although high-performance liquid chromatography was used to measure serum carotenoid levels, variations in sample collection, storage, transportation, and recent dietary intake may introduce measurement errors. Despite strict quality control measures, these potential interferences cannot be entirely eliminated, which may affect the accuracy of the results to some extent.

Building on the findings of this study, future research should expand in multiple dimensions to further elucidate the relationship between serum carotenoids and inflammatory markers. Prospective cohort studies and multi-center research, combined with advanced techniques in cell and molecular biology, could provide a comprehensive understanding of the precise molecular mechanisms by which carotenoids exert anti-inflammatory and antioxidant effects. This would involve exploring cellular signaling pathways and gene expression regulation, laying the groundwork for innovative applications of carotenoids in precision medicine and personalized nutrition.

## 5. Conclusion

Based on the NHANES database, this study thoroughly investigated the association between serum carotenoids and SII. The results conclusively demonstrate a significant negative correlation, which remains robust after rigorous adjustment for confounding factors and validation through multidimensional analyses. These findings further support the anti-inflammatory properties of carotenoids. Additionally, our study highlights the potential role of carotenoids in modulating systemic inflammatory states, offering new insights into their value in disease prevention and treatment. This research also provides novel theoretical evidence for understanding the mechanisms underlying systemic inflammation regulation.

## Acknowledgments

We thank Weijun Zheng’s team who provides the online analytical software for this study (www.zstats.net).

## Author contributions

**Conceptualization:** Hongcun Sun.

**Data curation:** Hongcun Sun.

**Supervision:** Jiandao Hu.

**Visualization:** Wenbo Jiang.

**Writing – original draft:** Hongcun Sun.

**Writing – review & editing:** Wenbo Jiang.

## Supplementary Material


