# Supplementary material for: Significant association of serum carotenoids with the systemic immune-inflammation index: A cross-sectional study based on NHANES
Source: Medicine (Baltimore). 2025 Jun 20;104(25):e42942. doi: 10.1097/MD.0000000000042942 (PMC12187323; doi:10.1097/MD.0000000000042942)
Supplement: Supplementary file 1 [file medi-104-e42942-s001.doc]

Supplementary Table 1 Association of serum carotenoids with SII after multiple imputation

| Variables | Model 1 | | Model 2 | | Model 3 | |
| --- | --- | --- | --- | --- | --- | --- |
|  | β (95% CI) | *P* | β (95% CI) | *P* | β (95% CI) | *P* |
| Total carotenoids | -0.77 (-0.91 ~ -0.64) | < 0.001 | -0.75 (-0.89 ~ -0.61) | < 0.001 | -0.61 (-0.75 ~ -0.47) | < 0.001 |
| α-Carotene | -3.33 (-4.17 ~ -2.49) | < 0.001 | -3.41 (-4.25 ~ -2.57) | < 0.001 | -2.78 (-3.63 ~ -1.93) | < 0.001 |
| β-Carotene | -0.92 (-1.17 ~ -0.67) | < 0.001 | -1.14 (-1.40 ~ -0.88) | < 0.001 | -0.93 (-1.19 ~ -0.66) | < 0.001 |
| β-Cryptoxanthin | -2.22 (-2.81 ~ -1.63) | < 0.001 | -1.94 (-2.55 ~ -1.32) | < 0.001 | -1.37 (-1.99 ~ -0.75) | < 0.001 |
| Lutein/zeaxanthin | -3.24 (-3.77 ~ -2.72) | < 0.001 | -2.80 (-3.34 ~ -2.26) | < 0.001 | -2.43 (-2.97 ~ -1.88) | < 0.001 |
| Lycopene | -1.48 (-2.02 ~ -0.93) | < 0.001 | -0.93 (-1.50 ~ -0.36) | 0.001 | -0.51 (-1.08 ~ 0.05) | 0.075 |

SII, systemic immune-inflammation index; CI, confidence interval.

Model 1: No variables was adjusted.

Model 2: Gender, age, race, educational level, PIR, and marital status were adjusted.

Model 3: All variables in Model 2 plus general health condition, BMI, smoking, alcohol use, vigorous activity, moderate activity, and CRP were adjusted.
